# Intervention in Mothers and Newborns to Reduce Maternal and Perinatal Mortality in 3 Provinces in South Africa Using a Quality Improvement Approach: Protocol for a Mixed Method Type 2 Hybrid Evaluation

**DOI:** 10.2196/42041

**Published:** 2023-06-05

**Authors:** Terusha Chetty, Yages Singh, Willem Odendaal, Solange Mianda, Nada Abdelatif, Samuel Manda, Helen Schneider, Ameena Goga

**Affiliations:** 1 HIV and Other Infectious Diseases Research Unit South African Medical Research Council Durban South Africa; 2 Discipline of Public Health Medicine School of Nursing and Public Health University of KwaZulu-Natal Durban South Africa; 3 Department of Psychiatry Stellenbosch University Cape Town South Africa; 4 South African Medical Research Council/University of the Western Cape Health Services to Systems Research Unit School of Public Health University of the Western Cape Cape Town South Africa; 5 Biostatistics Unit South African Medical Research Council Durban South Africa; 6 Department of Statistics University of Pretoria Pretoria South Africa; 7 Department of Paediatrics and Child Health University of Pretoria Pretoria South Africa

**Keywords:** health systems, quality improvement, antenatal, postnatal, maternal, child, HIV, mixed methods evaluation

## Abstract

**Background:**

The COVID-19 pandemic undermined gains in reducing maternal and perinatal mortality in South Africa. The Mphatlalatsane Initiative is a health system intervention to reduce mortality and morbidity in women and newborns to desired levels.

**Objective:**

Our evaluation aims to determine the effect of various exposures, including the COVID-19 pandemic, and a system-level, complex, patient-centered quality improvement (QI) intervention (the Mphatlalatsane Initiative) on maternal and neonatal health services at 21 selected South African facilities. The objectives are to determine whether Mphatlalatsane reduces the institutional maternal mortality ratio, neonatal mortality rate, and stillbirth rate (objective 1) and improves patients’ experiences (objective 2) and quality of care (objective 3). Objective 4 assesses the contextual and implementation process factors, including the COVID-19 pandemic, that shape Mphatlalatsane uptake and variation.

**Methods:**

This study is an implementation science type 2 hybrid effectiveness, controlled before-and-after design with quantitative and qualitative components. The Mphatlalatsane intervention commenced at the end of 2019. For objective 1, intervention and control facility-level data from the District Health Information System are compared for changes in institutional maternal and neonatal mortality and stillbirth rates and associations with QI, the COVID-19 pandemic, and both. This first analysis includes data from 18 facilities, regardless of their allocation to intervention or comparison, to obtain a general idea of the effect of the COVID-19 pandemic. For objectives 2 to 3, data collectors abstract data from maternal and neonatal records, interview participants, and conduct neonatal facility assessments. For objective 4, interviews, program documentation, surveys, and observations are used to assess how contextual factors at the macro-, meso-, and microlevels explain variation in intervention uptake and outcome. The intervention dose is measured at the microlevel only in the intervention facilities. The study assesses the Mphatlalatsane Initiative from 2020 to 2022.

**Results:**

From preliminary analysis, across the 3 provinces, maternal and neonatal deaths increased during the COVID-19 pandemic, whereas stillbirths remained unchanged. Maternal satisfaction with quality of care was >90%. The COVID-19 pandemic severely disrupted the QI teams functioning. However, the QI teams regained their pre–COVID-19 momentum by adapting the QI model, with advisers providing mentoring and support. Variation in adoption at the mesolevel was related to stable and motivated leadership (particularly at the facility level), poor integration into routine processes, and buy-in from senior district managers who were affected by competing priorities. Varying referral and specialist outreach systems, staff availability and development, and service delivery infrastructure are plausible factors in variable outcomes.

**Conclusions:**

Few evaluations rigorously evaluated the effect of health system interventions on improving health services and outcomes. Results will inform the scaling up of successful intervention components and strategies to mitigate the effects of the COVID-19 pandemic or similar emerging epidemics on maternal and neonatal mortality.

**International Registered Report Identifier (IRRID):**

DERR1-10.2196/42041

## Introduction

### Background

The emergence of COVID-19 caused by SARS-CoV-2 in 2020 had a detrimental effect on maternal and infant health gains. Pregnant and breastfeeding women are at a high risk for COVID-19–related morbidity and mortality [1,2]. Given the high number of pre–COVID-19 medically complicated pregnancies in low- and middle-income countries, the pandemic substantially increased the risks to pregnant and breastfeeding women and their infants [3] and undermined gains made in reducing maternal and perinatal mortality [4].

South Africa invested in improving sexual, reproductive, maternal, and neonatal health (SRMNH) services but did not reach the Millennium Development Goals targets for maternal and child health by the end of 2015. Aligned with the United Nations Sustainable Development Goals (SDG) of reducing maternal mortality to <70 per 100,000 live births and neonatal mortality to 12 deaths per 1000 live births [5], South Africa aims to reduce the institutional maternal mortality ratio (iMMR), neonatal mortality, and stillbirths by 50% by 2030 [6]. In 2019, the iMMR of 120 per 100,000 live births was well above the SDG target [6]. Moreover, although the institutional neonatal mortality rate (12 per 1000 live births in 2021) meets the SDG, almost 50% of neonatal deaths are avoidable (2021) [6]. This is despite the substantial reduction in iMMR since 2011, attributable to South Africa’s antiretroviral treatment program, which increased access to lifelong maternal antiretroviral treatment at higher CD4 cell counts [7].

National policies and strategies for SRMNH have been in place but lack a cohesive quality improvement (QI) strategy. These challenges have been exacerbated by the COVID-19 pandemic, which has complicated access to health care and delivery of maternal and perinatal interventions [4]. Furthermore, despite the strengths of South Africa’s existing maternal, perinatal, and child mortality audits, the availability of real-time data to address frontline challenges is undermined by outdated software and fragmented systems with inherent data quality issues and data gaps. These data gaps became more apparent as the pandemic necessitated rapid access to real-time maternal and perinatal data on COVID-19.

Optimal health care provides quality services that are effective and safe while also being timely, equitable, integrated, and efficient [8], with a focus on people-centered care as an essential component [9]. Quality of care is defined as the extent to which health services can increase the probability of desired health outcomes [10].

QI interventions in low- and middle-income countries have improved access to care, skilled delivery, and viral load monitoring. In a South African stepped-wedge randomized controlled trial, a continuous QI intervention resulted in a notable rise in viral load monitoring (relative risk [RR] 1.38, 95% CI 1.21-1.57; *P*<.001) [11]. However, there was no improvement in repeat HIV testing (RR 1.00, 95% CI 0.88-1.13; *P*=.96). In Ethiopia, there was a favorable effect on health workers’ adherence to safe childbirth practices immediately after birth (β=8.22, 95% CI 5.15-11.29) [12]. In Ghana, a QI intervention to reduce waiting time for emergency obstetric care and hand hygiene in the neonatal intensive care unit (ICU) improved hand hygiene compliance by 93%. Moreover, the percentage of mothers needing emergency cesarean surgery with unacceptable waiting times decreased by 4 times, and 93% of the sickest mothers were correctly identified [13].

Conversely, in 2 randomized controlled trials conducted at the primary care level in Nigeria and Malawi, continuous QI was ineffective [14,15]. The researchers rigorously measured continuous QI in terms of impact on neonatal and perinatal mortality rates in the Malawi study [14] and 6-month postpartum retention in the Nigerian study [15]. However, these end points do not reflect the QI activities that occurred in the primary care centers. The variability of these end points is likely related to elements outside the remit of the QI, such as emergency care availability, road infrastructure, and mothers’ educational attainment.

In 2017, the Mphatlalatsane Initiative was launched to provide a national unified response to improve the quality and equity of SRMNH care at the cusp of the National Health Insurance reform, using the Institute for Health Improvement QI methodology [16]. The final output is anticipated to be a proven and replicable model of catalytic health systems and clinical interventions to reduce unintended pregnancies and maternal and newborn deaths. These can be tested for sustainability and scaled up to emulate achievements across the rest of the country.

In this study, we report the protocol for the evaluation of the Mphatlalatsane Initiative. The initial aim of the protocol was to determine if a complex area–based QI initiative would reduce iMMR, neonatal mortality, and stillbirth rate by 50% in 5 years. Given the emergence of COVID-19 in March 2020 in South Africa with concomitant increased public health measures and national level-5 lockdown [17], the Mphatlalatsane Initiative was adapted to provide the much-needed support for maternal and perinatal care during the pandemic. The evaluation protocol was reframed to account for these adaptations and to determine the effect of the Mphatlalatsane Initiative on maternal and perinatal health care and outcomes, implementation processes, and health care system functioning during the pandemic from 2019 to 2022.

### Study Aims and Objectives

The evaluation primarily aims to measure the effectiveness of the Mphatlalatsane Initiative on iMMR, neonatal mortality rate, and stillbirth rate (objective 1). We also assess whether patients’ experiences of care (objective 2) and quality of maternal (objective 3a) and neonatal care (objective 3b) were affected by the pandemic. Objective 4a explores the macrolevel (regional and national) and mesolevel (subdistrict and district) factors, and objective 4b explores the microlevel (facility) process and contextual factors, including the COVID-19 pandemic, to explain variations in QI uptake and program outcomes and implications for scale-up. Partner interviews (objective 4a) commenced in early 2020, and data collection for objective 4b began in May 2021.

### Outcome Measures

The primary end points are (1) iMMR, (2) neonatal mortality rate, and (3) stillbirth rate. The secondary end points include patient experiences of health care quality and facility processes during the antenatal and postpartum periods (objective 2) and the quality of maternal (objective 3a) and neonatal care (objective 3b). The study also explores *macro-, meso-, and microlevel contexts* and implementation processes for improved quality and outcomes of maternal and neonatal health (MNH). Additional details on the secondary outcomes are provided in Multimedia Appendix 1.

## Methods

### Study Setting

The evaluation is underway in 4 intervention and 3 nonoverlapping comparison districts across 3 provinces: the Eastern Cape, Limpopo, and Mpumalanga. All catchment areas are in 1 district, except for the Eastern Cape intervention catchment area, which spans across 2 districts.

**Figure 1:**
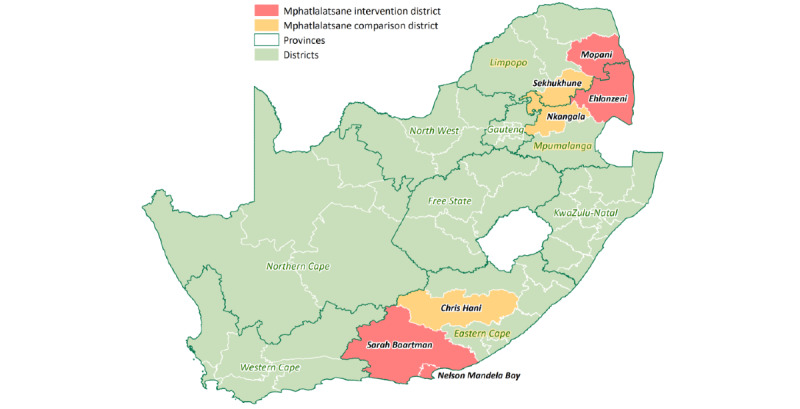
Mphatlalatsane intervention and comparison districts in the Eastern Cape, Limpopo, and Mpumalanga Provinces in South Africa. Produced by the Health GIS Centre of the South African Medical Research Council. Copyright ESRI 2016; ArcGIS Desktop: Release 10.4. Redlands, CA: Environmental Systems Research Institute.

The catchment area is subdivided into a wedge with 2 arms within each wedge. Each wedge has a district hospital at the apex. The hospital is linked to a regional hospital, 2 community health care centers, and 2 primary health care clinics in each arm. The intervention study districts in the Eastern Cape, Limpopo, and Mpumalanga are highlighted in red in Figure 1. The comparison facilities and the intervention facilities are in different districts to avoid contamination, and the former will not have the same apex district hospital as the intervention site. The comparison districts (Figure 1 in yellow) are not evaluated for objective 4.

### Study Design

We are conducting a mixed methods implementation science type 2 hybrid effectiveness design to study the intervention effect (objectives 1-3) and implementation (objective 4) during the COVID-19 pandemic. Given that the intervention facilities were preselected, the quantitative component will be implemented as a quasi-experimental controlled before-and-after study (nonrandomized assignment of the intervention and comparison groups) to measure the effect of a QI intervention implemented during the COVID-19 pandemic on maternal and neonatal outcomes. Data on the iMMR, neonatal mortality rate, and stillbirth rate will be analyzed from 2019 to 2021, including the time before and during the COVID-19 pandemic in South Africa (objective 1). Study data on maternal experiences of care (objective 2) and maternal and neonatal quality of care (objectives 3a and 3b) were collected between 2021 and 2022. Objective 4 data collection commenced in early 2020 and ended in August 2022.

### Eligibility Criteria and Recruitment of Facilities

#### Overview

The intervention facilities were purposively identified by the National Department of Health (NDOH) as sites with MNH service performance and capacity below the national average in South Africa. In addition, the selected intervention sites reflect the clinical and referral pathways and represent both urban and rural settings. For objectives 2 and 3, the comparison districts were purposively selected to match the intervention districts in as many respects as possible, including iMMR, stillbirth rate, neonatal mortality rate, sociodemographic characteristics, service organization, and setting (rural, urban, and periurban), and to provide the NDOH standard of care. In total, 3 facilities (2 district hospitals and 1 regional hospital) in the comparison district were selected and matched to the intervention district based on the total number of births. Data collection for objectives 2 and 3 is conducted in 18 hospitals (15 district- and 3 regional-level hospitals). Objective 4b is being conducted in 15 hospitals, purposively selected from the 21 intervention facilities.

#### Participant Inclusion Criteria for Maternal Interview Shortly After Delivery (Objective 2) and Data Abstraction From Maternity Records (Objective 3)

To assess patient experiences of care and determine the quality of maternal care, participants who are aged ≥18 years, has a pregnancy diagnosis, who accessed care within the participating health facilities during their pregnancy, or who are within 1 week post partum are eligible. Research assistants will screen for eligibility using specified inclusion criteria. All women meeting any of the following criteria will be eligible: (1) pregnant women at ≥36 weeks gestation without any complications, (2) pregnant women at ≥36 weeks gestation with HIV before pregnancy and on antiretroviral therapy and diagnosed with HIV at first antenatal visit, (3) positive repeat antenatal HIV test following the first negative antenatal HIV test, (4) pregnant women at ≥36 weeks’ gestation with eclampsia, (5) pregnant women at ≥36 weeks’ gestation with primary postpartum hemorrhage, (6) postpartum women at ≤7 days with a premature baby, (7) postpartum women at ≤7 days with a healthy term baby, and (8) postpartum women at ≤7 days with a baby in the ICU. The medical records of eligible cases will be retrieved for a thorough review at hospital discharge or on transfer to another hospital.

Data collectors will conduct daily visits to maternity wards to recruit maternal participants and determine the antenatal quality of care during the pandemic era. During the daily visit, the admission and maternity registers are screened for participants who meet the study inclusion criteria. Stable women with recent deliveries are approached by an interviewer for recruitment before leaving the facility and are interviewed in a private space to ensure confidentiality or via telephones if on-site interviews are not possible. Women are excluded from participation if they are aged <18 years, delivered at home or on the way to the facility, are acutely ill in the ICU or with life-threatening conditions, or are emotionally distressed. Women whose infants are admitted to the ICU or who experienced a stillbirth or neonatal death will be approached for interview, and if they agree, they will be interviewed at a time that suits the mother.

#### Participant Inclusion Criteria (Objective 4)

At the macrolevel (provincial and national) and mesolevel (subdistrict and district; objective 4a), the study team will purposively recruit subdistrict, district, and area managers and those specifically responsible for managing maternal and neonatal care services across subdistricts, districts, provinces, and areas. At the microlevel (facility; objective 4b), we include all QI advisers, that is, persons who provide QI expertise and support to participating facilities’ health workers who are organized in QI teams as well as the operational managers of the sampled facilities.

### Procedures

#### Conceptual Framework

Our initial hypothesis was that the QI methodology will enhance the quality of MNH care by creating an enabling environment for leadership and governance to flourish, ensuring that clinical processes are delivered safely and reliably. A key component of the QI approach is ensuring respectful care and support for patients. We further hypothesize that optimizing the environment and processes will improve the health care system, leading to improved health outcomes and patient experience of care. Our underlying causal assumptions are that QI will empower macro- and mesolevel management and microlevel health workers to drive and implement change.

Implicit assumptions are staff participation in QI training, a facility QI team that continues QI activities throughout study implementation, and a leader within that team with continuity of participation are integral to its success. At the start of the intervention, the Mphatlalatsane Initiative implemented the Institute for Healthcare Improvement model for improvement as a framework to guide its implementation strategy [10]. The strategy involves the identification of a specific goal for improvement and a plan to measure progress and then commences with change ideas that may lead to improvement over a short duration. As the change ideas are refined and successfully applied, the interventions are broadened to increase the magnitude of the changes. The Institute for Healthcare Improvement stresses initiating improvement on a small scale and leveraging the obtained knowledge to plan for wider implementation and eventual scale-up. Additional assumptions included that other competing programs or priorities implemented by the NDOH would not be conducted in the intervention catchment areas. However, the COVID-19 pandemic disrupted QI team functioning. The consortium partners prioritized the immediate needs of facilities, which broadened the initial intervention scope (described below under *Intervention Dose* section) of the Mphatlalatsane Initiative.

A framework of mesolevel (district and subdistrict) enabling environments was developed a priori, which included factors of distributed leadership and buy-in, area-based mechanisms of service delivery coordination and review, referral and outreach systems, and the allocation of resources in response to identified needs at the facility level.

#### Intervention Package

The Mphatlalatsane Initiative initially aimed to improve the quality of maternal and neonatal care in the participating facilities through QI activities. A QI adviser has been appointed in each catchment area to manage and support these teams. We anticipate that these teams will become nodes of “learning practices,” with team members pooling their efforts and skills toward solving targeted service problems [18]. We view the respective microlevel health systems, in which the QI teams function, as social systems, with the agency of staff and the relationships, existing and to-be-established, within these systems [19], as key to understanding the teams’ performance. The evaluation and intervention teams are independent to minimize bias. The intervention design of in-person support shifted based on facility responses during the COVID-19 pandemic. Initial attempts to use digital platforms to support QI teams were unsuccessful, and QI advisers subsequently supported QI teams through telephonic messages and calls. Other design changes included providing emotional support and guidance and improving the efficiency of supply chain management for infection control and personal protective equipment. Once the acute demands were met, the Mphatlalatsane intervention was revised to mitigate the interruptions in access to routine MNH services.

QI activities comprise the repeated and rapid implementation of Plan-Do-Study-Act (PDSA) cycles [20,21]. The *Plan* refers to problem identification and development of solutions; the *Do* refers to implementation of the solutions; the *Study* refers to the evaluation of effects of the solutions and then *Acting* on the outcome of the evaluation by adopting, adapting, or rejecting the solutions [20,21]. QI teams have been established in each participating facility to engage maternal and neonatal staff in relevant PDSA activities [21,22].

#### Comparator

Health care providers in the control district facilities continue to provide the NDOH standard of care to antenatal and postnatal attendees as per the policy and guidelines. The usual training for staff involves weekly 1-hour in-service training on current evidence-based guidelines. The usual training does not include a coaching or mentoring component and is not a data-driven process for evaluating the implementation of evidence-based guidelines.

### Data Sources

#### Objective 1

Mortality data from 2017 to 2022 were extracted from the District Health Information System (DHIS; Table 1).

**Table 1:** Mphatlalatsane data sources.

Objective	Data source	Variable or theme	Level of analysis
1	DHIS^a^	Mortality data (maternal, neonatal, and stillbirths) extracted quarterly from 2019 to 2022	District and facility
2	Participant interview shortly after delivery in regional or district hospitals	The effect of the QI^b^ intervention over time on patients’ experience of the quality of antenatal and postnatal health care services from 2021 to 2022	Individual
3a	Maternal records from regional or district hospitals	Facility audit of maternal records will be conducted to measure adherence to clinical protocols on normal pregnancy, HIV, eclampsia, and postpartum hemorrhage from 2021 to 2022	Individual
3b	Structured observation checklists will be interviewer administered in selected facilities based on the Newborn QI Toolkit norms and standards for newborn care, which were developed to collect data in each facility [23]	Availability of equipment and supplies Availability of guidelines or policies Distribution and availability of drugs Data quality and use Staffing Infrastructure	Facility
4a	Semistructured interviews and FGDs^c^ with the QI advisers, operational managers, and management staff	Stakeholder analysis: coordination processes, nature and quality of social networks, decision-making processes, formal and informal communication, and collaboration between key actors. We will set up monthly individually open-ended debriefing meetings with the QI advisers, and they will reflect on their work and engagement with the QI teams. We will also review program documentation, including the documentation that the QI teams will generate during the PDSA^d^ cycles. We will also conduct other program meetings at the macro- and mesolevels.	National, provincial, district and subdistrict
4b	Interviews and FDGs with QI teams QI team surveys QI adviser debriefings Program documentation	QI advisers, teams, and facility managers: setting up, management structures, systems of communication, translation of their PDSA cycles into everyday practices, climate for QI activities, relational dynamics in the teams, ownership of change, resource use, experiences, and perceptions of implementation changes over time, perceived benefits, and challenges of the QI work in their facilities. QI team outcomes: packaging learning practices for scale-up and identify the “leverage points” for change.	Facility

^a^DHIS: District Health Information System.

^b^QI: quality improvement.

^c^FGD: focus group discussion.

^d^PDSA: Plan-Do-Study-Act.

#### Objective 2

In-person or telephonic interviews were conducted with pregnant women shortly after delivery to measure participants’ subjective experiences of care. The survey used a 5-point Likert scale based on a prior study of maternal patient satisfaction with services in Tanzania [24]. Data collectors administered paper-based surveys at the midline, which were then captured using REDCap (Research Electronic Data Capture; Vanderbilt University) software.

#### Objective 3a

Data collectors took photographs or abstracted data from maternal records, which were then entered into the REDCap database. Facility audit of maternal records were conducted by an experienced nurse to measure adherence to the guidelines and implementation of care. Data were collected at a single time point within 1 week of delivery.

#### Objective 3b

Interview-administered structured observation checklists were completed in all selected facilities based on the Newborn QI Toolkit [23] norms and standards for newborn care.

Data were also collected using a series of checklists to determine the quality of record keeping for the newborns. Staff members in the neonatal unit were requested to provide the clinical records of a sample of newborns with the following conditions: (1) low birth weight, (2) infection, and (3) asphyxia. A review of these records was conducted to determine the quality of record keeping. Data collection for objective 3 was at the end line (approximately June 2022 to August 2022).

#### Objective 4a

Semistructured interviews and focus group discussions (FGDs) were conducted with the QI advisers, QI teams, operational managers, and management staff at the subdistrict, district, and provincial levels.

#### Objective 4b

The data collection comprised (1) interviews and FGDs with the QI team leaders and members, (2) digital surveys to leaders and teams to assess their performance, (3) regular debriefings with the advisers, and (4) a review of program documentation. The debriefings and documentation reviews commenced in February 2020, and the interviews, FGDs, and surveys began in May 2021. As of November 2022, we conducted 32 interviews with leaders and 51 interviews or FGDs with team members.

### Sample Size

#### Objective 1

Using the number of deliveries and maternal and perinatal deaths in 9 regional and district facilities in the chosen intervention catchment areas (the Eastern Cape, Limpopo, and Mpumalanga) between 2018 and April 2019, rates were found to be 115.4 maternal deaths per 100,000 live births, 14.4 neonatal deaths per 1000 live births, and 22.5 stillbirth births per 1000 facility births. For this study, an intraclass correlation estimator from a random intercept logistic regression model was used. Using the average facility size (based on deliveries in the intervention catchment areas during the baseline period), the variance inflation factor was estimated at 1.0, 1.4, and 1.3 for the maternal mortality ratio, neonatal mortality rate, and stillbirth rate, respectively. To test the hypothesis that there was no improvement against a 50% increase in the baseline, with 80% power at a 5% two-sided significance level, the number of live births would be 40,524 and 4512 for testing maternal and neonatal mortality rates and 2666 for stillbirth rates. We will observe at least 41,000 deliveries pooled across the 3 catchment areas over a 3-year period to have minimum power for all outcome comparisons. The same would accrue in the control facilities.

#### Objective 2 and 3a

Literature on patient satisfaction in South Africa is scarce; in other studies, patient satisfaction varies from <50% to as high as 75% [25]. The impact of the intervention on patient satisfaction may likely be less compared with that before the COVID-19 era because of disruptions in service delivery. For the 18 facilities in the intervention arm and 18 facilities in the control arm, at a power of 80%, and an expected increase in patient satisfaction from 50% to 60%, given an intraclass correlation coefficient of 0.06, the number of pregnant women required is 102, with a total sample size of 1529 per arm in the pre- and postintervention periods.

#### Objective 3b

The study team will evaluate 18 facilities (3 in each of the intervention and control catchment areas).

#### Objective 4

Sampling at the meso- and macrolevels will be linked to the microlevel sampling. The subdistricts and districts where facilities have been selected and their respective area levels will be included in the evaluation at the macro- and mesolevels. At minimum, 2 subdistricts, 2 districts, and 3 areas (total 7 “units” of assessment) will be selected, although additional units could be added if primary health care clinics, community health care centers, and district hospitals are not all from the same subdistrict. We anticipate approximately 90 and 120 interviews or FGDs, respectively, for the macro-, meso-, and microlevel interviews or FGDs over the 18 months of data collection. At the microlevel, we sampled QI teams across the 3 catchment areas, as follows:

Objective 4b facilities (Table 2) were sampled based on readiness assessment scores from the NDOH (this was done before the start of the intervention to assess the state of maternal and neonatal care in the intervention facilities) and input from the QI advisers and represent the range of facility types; MNH care packages; and urban, semiurban, and rural areas.

**Table 2:** Type of objective 4b facilities sampled in Eastern Cape, Limpopo, and Mpumalanga from 2020 to 2022 (N=15).

	Eastern Cape (n=4), n (%)	Limpopo (n=5), n (%)	Mpumalanga (n=6), n (%)
Regional hospital (n=3)	1 (7)	1 (7)	1 (7)
District hospital (n=4)	1 (7)	1 (7)	2 (13)
Community health care center (n=5)	1 (7)	2 (13)	2 (13)
Primary health care clinic (n=3)	1 (7)	1 (7)	1 (7)

### Intervention Dose

The intervention dose, defined as the “quality and quantity of an intervention and participation” [26], may be necessary to determine the barriers or enablers of the Mphatlalatsane Initiative. However, measuring the intervention dose is complex and requires substantial data collection to quantify the various intervention dose dimensions. Moreover, there are currently no accepted methods for measuring this dose. McHugh et al [26] outlines 8 dimensions of intervention dose that may affect whether or not a QI initiative succeeds. In the Mphatlalatsane Initiative, the measurement of the intervention dose is further complicated by the COVID-19 pandemic. The implementation of the Mphatlalatsane Initiative was ongoing for approximately 6 months when the first case of COVID-19 was diagnosed in South Africa in March 2020 [27]. Following the national state of the disaster and level-5 lockdown, the Mphatlalatsane Initiative was redefined to provide support to the intervention sites. This support took the form of a web-based platform, telephonic meetings, and in-person QI adviser support when lockdown restrictions eased. However, the use of the web-based platform to provide support was unsuccessful owing to issues with internet connectivity, the lack of equipment, and user competence. As such, the Mphatlalatsane Initiative implementation may have varied at the micro-, meso-, and macrolevels. To determine the Mphatlalatsane intervention dose at the microlevel, a scoring system is in development based on prior work [26]. The first 2 domains, exposure and intensity, relate to the QI adviser. *Exposure* is the number of interactions between the adviser and the QI team; *intensity* is the nature of the interaction between QI advisers and QI teams, for instance, face-to-face support to the team in reviewing their change ideas and offering technical advice during the support visit (high intensity) or a mobile call from the adviser to find out when the team will send their QI documentation (low intensity). The remaining 4 domains relate to the adopted change idea. Quantity is the number of adopted (ie, change ideas with quantifiable evidence that it improves care) change ideas; *reach* is the number of facility workers involved; *duration* is the implementation period in months; *scope* is the 5 Mphatlalatsane outcomes that have been targeted for the change ideas in terms of leadership, clinical care, health systems, patients’ experience of care, and patients’ health. For the period from September 2019 to December 31, 2021, retrospective data are collected by QI advisers for 4 domains: quantity, reach, duration, and scope (Multimedia Appendix 2). For the period from January 1 to December 31, 2022, all domains will be extracted from the adviser’s routine reports (Multimedia Appendix 2). The scoring of each domain and assessment of the intervention dose are evolving and will be finalized once the data have been collected. It is envisaged that 2 members of the evaluation team will independently score each domain, and disagreements will be resolved by a third member.

### Statistical Analysis

#### Objective 1

The institution maternal and perinatal mortality indicators will be calculated using DHIS data. Trend in iMMR, neonatal mortality rate, and stillbirth rates (with SE bars) during the pre- and postintervention periods will be disaggregated by intervention arms. RR and 95% CI will be calculated along with data on confounding variables, such as antenatal care coverage before 20 weeks, delivery at health facility access by tar road, deliveries of female participants aged <19 years, and deliveries of female participants aged ≥19 years (derived from total deliveries and deliveries of female participants aged <19 years). The trends in mortality and stillbirths will be compared before the COVID-19 pandemic (January 2019-March 2020) and during the COVID-19 pandemic (April 2020-March 2021) and in the context of the COVID-19 waves. The baseline period (2017-2018) will also be provided. Observed numbers and rates will be determined monthly and quarterly across all 9 provinces in the country and overall. To compare the Mphatlalatsane Initiative intervention and control arm trends in mortality, an interrupted time series regression model using Poisson regression model with Newey-West SEs to account for autocorrelation and possible heteroskedasticity will be used.

#### Objective 2

This study will use quantitative descriptive analyses and exploratory factor analysis to examine associations between sociodemographic characteristics, childbirth characteristics, and maternal participants’ experience of health care quality and the health care facility during the antenatal to postpartum period.

#### Objective 3a and 3b

Quantitative data analysis will be conducted on demographic data as well as specific management elements offered to participants with hypertension in pregnancy and primary postpartum hemorrhage based on predetermined criteria [28]. Patients with normal pregnancies will be scored against the NDOH clinic checklist for booking and follow-up of patients without HIV [29]. A study nurse will allocate the score based on antenatal and intrapartum data abstracted from maternity records. The scoring system will be as follows: for any variable that is fully met, 1 unit will be awarded; if partially met, half of a unit will be awarded; whereas if absent, 0 will be awarded. If a patient is managed for more than one of the specific complications, each entity will be assessed separately. The criteria scores for each patient will be individualized. The level of care for each complication will then be assessed as follows:

*Care score/Criteria score × 100*
**(1)**

and is similar to a prior study evaluating QI in neonatal services in KwaZulu-Natal, South Africa [30].

#### Objective 4

Assuming that attitudes will be complex, grounded theory will be used during this research to keep an open view. Instead of beginning with hypotheses about attitudes, a theory will be developed during the data collection. The interviews and FGDs will be analyzed with qualitative data analysis by using coding, concepts, and categories to develop a theory. The interviews and FDGs will be recorded and transcribed using Microsoft Word (Microsoft Corp). First, a South African Medical Research Council (SAMRC) and a University of Western Cape (UWC) staff member will read through all texts, look for patterns, and assign codes for these patterns. These codes will be assigned to all texts using a qualitative data analysis software program, Atlast.ti (version 8; ATLAS.ti Scientific Software Development GmbH). The SAMRC and UWC staff will then jointly describe these codes and develop categories from combinations of codes and triangulation of concurrent quantitative and qualitative data and then develop hypotheses based on relationships between these concepts, which will form the theoretical framework. The survey data will be imported into Excel, and descriptive analysis techniques will be used to analyze the data.

All quantitative data will be entered into an electronic format and stored in a web-based interface. The quantitative questionnaires will be analyzed using Stata (version 16; StataCorp) [31].

### Ethics Approval

#### Overview

This study has been approved by the SAMRC Human Research Ethics Committee (EC019-11/2019). The proposal for objective 4 was approved by the UWC Biomedical Research Ethics Committee (BM19/10/16). All participants sign written informed consent or provide verbal consent when the participant is illiterate, with an independent witness present to confirm that the relevant information was understood by the participant.

#### Objectives 1 to 3

We inform all patients and staff about the evaluation and elicit written informed consent before all interviews, photographs, and data abstraction from the maternity registers and maternity records. Data are deidentified before photographs are taken, and the interview takes approximately 30 minutes with participant confidentiality maintained. Survey administration entails minimal risks to patients and health staff. The results are anonymous and kept confidential. Although participants may decline the interview, we obtained permission from the NDOH to access and analyze data from the DHIS from the relevant catchment areas of the Eastern Cape, Limpopo, and Mpumalanga. We also have approval from the National and Provincial Department of Health to access routine records and photograph maternity case records.

#### Objective 4

##### Recruitment of Participants: Interviews, FGDs, and Observations

Written informed consents are obtained from all the individuals who participate in the interviews, FGDs, and observations. A detailed explanation of the purpose and nature of the study is provided to all the participants verbally and in writing with the information sheet in English. For the digital survey, participants receive the same study information as for the other data collection components and provide a digital signature to prove their consent.

Voluntary participation and the right to withdraw from the study at any point is emphasized during this explanation. Participants can consider their participation and ask questions before signing the informed consent form. Data collection occurs at a time and place that is convenient for the participants to ensure that health care delivery is not disrupted. Although anonymity cannot be guaranteed in FGDs, participants are requested to treat the information discussed as confidential. Participants who complete the surveys using personal devices are reimbursed with a data voucher. The NDOH requested that the interview and FGD participants not be reimbursed, given that they participate in working hours.

##### Accessing Facility Service Data Used by the QI Teams

Permission to access these data has been obtained from the NDOH and the respective Provincial Departments of Health.

## Results

### Institutional Live Births, Maternal Deaths, Neonatal Deaths, and Stillbirths

Data from the DHIS in the Eastern Cape, Limpopo, and Mpumalanga revealed 1,093,372 live births during the pre–COVID-19 period from March 2019 to March 2020 versus 1,037,528 during the COVID-19 pandemic (April 2020-March 2021). There were 1024 maternal deaths reported from March 2019 to March 2020 versus 1315 maternal deaths during the COVID-19 pandemic. There was a small decline in neonatal deaths from 13,095 during the pre–COVID-19 period to 13,026 deaths during the COVID-19 pandemic. The number of stillbirths also decreased from 22,030 during the pre–COVID-19 period to 20,891 during the COVID-19 pandemic.

### Maternal Satisfaction With Care

From October 27, 2021, to July 8, 2022, there were 3934 completed maternal interviews across 18 hospitals. In the maternal interviews, participants were largely satisfied with their antepartum care (3556/3841, 92.58%). Approximately 5.88% (226/3841) of the participants self-reported complicated deliveries.

### Microlevel Contexts and Implementation Processes

In the preliminary analysis, although the COVID-19 pandemic severely disrupted the functioning of the QI teams, the pre–COVID-19 momentum was regained, with advisers providing mentoring and support and teams adapting the QI model.

### Mesolevel (Subdistrict and District) Enabling Environments

Variation in adoption was related to stable and motivated leadership (particularly at the facility level) and buy-in from senior district managers (affected by competing priorities and a lack of integration into routine processes). Referral and specialist outreach systems, staff availability and development, and service delivery infrastructure also varied, which are plausible factors in variable outcomes.

## Discussion

### Overview

In our preliminary results, the routine mortality data on maternal and neonatal deaths and stillbirths reflected a mixed picture regarding maternal and perinatal care in South Africa before and during the COVID-19 pandemic. Across the intervention and control facilities, the participants were satisfied with the antepartum and intrapartum care received.

The increase in institutional maternal and neonatal deaths from the Eastern Cape, Limpopo, and Mpumalanga is consistent with prior evidence from Pattinson et al [4] and Pillay et al [32], who note that increased maternal and neonatal mortality was a direct effect of COVID-19–related deaths in pregnant women and an indirect effect of poor access to health services during the pandemic. This study reports on 3 provinces where the Mphatlalatsane Initiative was implemented versus the national reports from Pattinson et al [4] and Pillay et al [32]. Stillbirth rate appeared to remain relatively unchanged during the COVID-19 pandemic in these provinces. The major data limitation is the reliance on a single data source, the DHIS. Further analyses will stratify the catchment areas according to intervention and control facilities. The preliminary objective 4b data point to important contextual and implementation process differences across districts; for instance, a travel embargo in 1 district restricted the district management support visits to the intervention facilities, which have been reported by other districts to be an important motivation for QI teams. We are also observing differences between facilities in the same district, for example, some leaders are more motivated to implement the intervention than others. Unpacking the contextual and implementation processes that are shaping the QI intervention may facilitate the understanding of the Mphatlalatsane Initiative outcomes.

Globally, health systems have faced unprecedented strain and uncertainty during the COVID-19 pandemic [33]. Policy makers and health care providers made rapid shifts in all aspects of the health system to mitigate the COVID-19 risk [33] and accommodated the growing COVID-19–related morbidity and mortality. These changes included performing remote patient consultations [34]; repurposing wards and hospitals for COVID-19 [34]; and developing innovations, including vaccines [35], to counter the COVID-19 risk.

Several authors have suggested ways in which QI might be applied during the pandemic; for instance, rapid learning collaboratives, which are the most successful interventions when changes are occurring concurrently [36]. QI could enhance the health system, provide a methodology for systematic change, and improve learning [37,38].

When a swift response to changing circumstances is required, rapid learning cycles, such as through the PDSA structure, can enable teams to adapt quickly with minimal risk and interruption to clinical work. Having a mechanism to try, and refine, ideas and ensuring that they are plausible before implementing them can support teams in responding to challenges that have no known solution, as described by Fitzsimons [36].

This evaluation protocol aims to determine the effect of the Mphatlalatsane Initiative on maternal and perinatal health during the COVID-19 pandemic. A key strength of this evaluation is that the study is embedded within the public health system and implemented within routine structures, processes, and data and importantly within available human resources. Moreover, the QI initiative ran for approximately 6 months before the pandemic arose in March 2020 in South Africa. Thus, it is possible to evaluate how various exposures, including the COVID-19 pandemic and the Mphatlalatsane Initiative, affected maternal and perinatal care in the intervention districts. In particular, the evaluation will consider the COVID-19 burden of disease in intervention and control districts and the heterogeneity in maternal and perinatal care during and between COVID-19 waves. As the Mphatlalatsane intervention broadened from the QI methodology to supporting MNH health services during the COVID-19 pandemic [16], we cannot discount the effect of the pandemic when evaluating the causality between the Mphatlalatsane intervention and MNH outcomes. Moreover, the process evaluation will capture the nuanced changes that were occurring as the Mphatlalatsane Initiative was evolving and the iterative way the intervention was delivered with changes responsive to health system requirements (adaptive *function*).

### Limitations

This study has some limitations. As mortality data are extracted from the DHIS, which contains summary-level data on maternal and neonatal deaths and stillbirths, we cannot account for missing or incomplete data. A further limitation is that the QI intervention dose was not equally applied across facilities in the chosen districts, as the pandemic interrupted QI training and support structures that were necessary to implement the intervention. Although the evaluation team will strive to measure the intervention dose in a structured manner, the complex manner in which the intervention adapted to the requirements of the health system, particularly during the COVID-19 pandemic, may mean that only a few dimensions of the intervention dose may be captured in the analysis [26]. Moreover, the QI advisers collected intervention dose data both retrospectively and prospectively, which limits interpretation. There were also several Mphatlalatsane staff changes during the implementation period, which further limited the collection of the intervention dose data. Furthermore, data on policy and change at the macro- and mesolevels may not be able to draw narrow causal relationships between the Mphatlalatsane Initiative and outcomes at the facility (micro) level. However, qualitative data from the macro-, meso-, and microlevels may provide a more nuanced picture of the complexity of the Mphatlalatsane Initiative.

The results of the Mphatlalatsane evaluation will inform whether the Mphatlalatsane Initiative in the context of the COVID-19 pandemic is effective at improving maternal and neonatal mortality and stillbirth rate in a resource-limited setting. The results will inform strategies to mitigate the effects of the COVID-19 pandemic or similar emerging epidemics on maternal and neonatal mortality.

## Appendix

Multimedia Appendix 1 Mphatlalatsane secondary outcomes.

Multimedia Appendix 2 Mphatlalatsane quality improvement scorecard.

## Abbreviations

DHIS: District Health Information System
FGD: focus group discussion
ICU: intensive care unit
iMMR: institutional maternal mortality ratio
MNH: maternal and neonatal health
NDOH: National Department of Health
PDSA: Plan-Do-Study-Act
QI: quality improvement
REDCap: Research Electronic Data Capture
RR: relative risk
SAMRC: South African Medical Research Council
SDG: Sustainable Development Goals
SRMNH: sexual, reproductive, maternal, and neonatal health
UWC: University of Western Cape

## Data Availability

The data sets generated and analyzed during this study are not publicly available because some data are owned by the South African National Department of Health but are available from the corresponding author on reasonable request.
