# Supplementary material for: Intervention in Mothers and Newborns to Reduce Maternal and Perinatal Mortality in 3 Provinces in South Africa Using a Quality Improvement Approach: Protocol for a Mixed Method Type 2 Hybrid Evaluation
Source: JMIR Res Protoc. 2023 Jun 5;12:e42041. doi: 10.2196/42041 (PMC10280336; doi:10.2196/42041)
Supplement: Multimedia Appendix 1 [file resprot_v12i1e42041_app1.docx]

## **S1 Appendix: Secondary Outcome Measures**

Objective 2:

1. Patient experiences of health care quality and facility processes during the antenatal, intrapartum, and postpartum period.

Objective 3a: Maternal health outcomes

1. HIV retesting three-monthly during pregnancy and at delivery.
2. ART initiation in HIV-infected women preconception, during pregnancy, and after delivery.
3. Proportion of HIV-infected women with viral loads (VLs) documented (including VL date) in the maternity case record.
4. Proportion of HIV-infected women with VLs at delivery.
5. Maternal HIV virologic suppression among women who are HIV-infected.
6. Mean care score for normal pregnancy without complications.
7. Mean care score for postpartum haemorrhage; and
8. Mean care score for eclampsia.

Objective 3b: Quality of neonatal care

1. Compliance with items contributing to the resources score.
2. Compliance with items contributing to the resuscitation score.
3. Compliance with items contributing to the newborn records score; and
4. Composite quality of care score.

Objective 4a: Macro and meso level contexts and implementation processes of an enabling sub-district, district, provincial and regional environment for improved quality and outcomes of MNH, specifically:

1. The nature and extent of distributed leadership for MNH.
2. The functioning of governance structures and processes (planning, coordination, accountability, decision making, ownership, support) for improved MNH.
3. Availability of resources, and referral systems for MNH; and
4. The role of contextual factors in shaping the implementation of the interventions, and conversely, the role of the interventions in shaping contextual factors.

Objective 4b: Micro level contexts and processes

1. Describing the contexts, interactions, and implementation processes of QI teams in participating facilities.
2. Assessing how the elements under a) shape how QI teams’ function and are managed, as units within themselves, and as part of the facility; and
3. Evaluating team leadership within QI teams, describing how this develops over time and assessing how it shapes team functioning.
