# Supplementary material for: Intervention in Mothers and Newborns to Reduce Maternal and Perinatal Mortality in 3 Provinces in South Africa Using a Quality Improvement Approach: Protocol for a Mixed Method Type 2 Hybrid Evaluation
Source: JMIR Res Protoc. 2023 Jun 5;12:e42041. doi: 10.2196/42041 (PMC10280336; doi:10.2196/42041)
Supplement: Multimedia Appendix 2 [file resprot_v12i1e42041_app2.docx]

**Mphatlalatsane evaluation: QI scorecard**

1. **Collecting historical QI intervention data**

*To be completed by the QI advisors for each change idea that has been adopted and implemented between Sept ’19 and Nov ’21.*

**QI advisor:** ______________________________ **Date:** ___________________

**Facility name:** _______________________________________________________________________

**Adopted change idea title:** ______________________________________________________

1. **Reach:** The number of facility workers, clinicians, and non-clinicians, involved in implementing the adopted change idea

Clinicians (nurses, doctors): _________

Non-clinicians (for example community health workers, security guards, information management):

_________

Total: _________

Any additional comments from Advisor: ________________________________________

1. **Duration:** The calendar months which the adopted change idea has been actively implemented:

_________ months, from _______ /________ (month / year) to _______ /________ (month / year)

Any additional comments from Advisor: _______________________________________

1. **Scope:** Does the adopted change idea improve one or more of the following outcomes (mark with X)

*Leadership:* Yes

No

Unsure, to discuss with Evaluation team

*Clinical care:* Yes

No

Unsure, to discuss with Evaluation team

*Health system issues:* Yes

No

Unsure, to discuss with Evaluation team

*Patient’s experience of care:* Yes

No

Unsure, to discuss with Evaluation team

*Patient’s health:* Yes

No

Unsure, to discuss with Evaluation team

Any additional comments from Advisor: ________________________________________

1. **Collecting data on prospective QI and other interventions (only *Intensity* applies to ‘other interventions’)**

*We kindly request that the information described in the six domains be made part of the Advisors’ routine reporting, starting 1 Dec ’21.*

1. **Quantity:** Record the start and end date for each change idea adopted since 01 Dec ’21
2. **Exposure:** Record the number of your interactions with the QI teams
3. **Reach:** The number of facility workers, clinicians, and non-clinicians, involved in implementing the adopted change idea
4. **Duration:** The calendar months which the adopted change idea has been actively implemented
5. **Intensity:** A short description of the type and nature of your interaction with the QI team, for instance: face-to-face visit and reviewing PDSA documentations, WhatsApp to encourage staff to submit reports, or a telephone call to help them with a problem. This includes non-QI interventions you initiate / are involved in, for instance helping with a health system issue such as referrals, supplies and commodities
6. **Scope:** Does the adopted change idea improve one or more of the following outcomes:

*Leadership, Clinical care, Health system issues, Patient’s experience of care, Patient’s health*
